# Small-Molecule Inhibitors Overcome Epigenetic Reprogramming for Cancer Therapy

**DOI:** 10.3389/fphar.2021.702360

**Published:** 2021-09-17

**Authors:** Wenjing Xiao, Qiaodan Zhou, Xudong Wen, Rui Wang, Ruijie Liu, Tingting Wang, Jianyou Shi, Yonghe Hu, Jun Hou

**Affiliations:** ^1^ School of Materials Science and Engineering, Southwest Jiaotong University, Chengdu, China; ^2^ Department of Pharmacy, The General Hospital of Western Theater Command of PLA, Chengdu, China; ^3^ Department of Ultrasonic, Sichuan Academy of Medical Sciences and Sichuan Provincial People’s Hospital, School of Medicine, University of Electronic Science and Technology of China, Chengdu, China; ^4^ Department of Gastroenterology and Hepatology, Chengdu First People’s Hospital, Chengdu, China; ^5^ Information Department of Medical Security Center, The General Hospital of Western Theater Command of PLA, Chengdu, China; ^6^ Personalized Drug Therapy Key Laboratory of Sichuan Province, Department of Pharmacy, Sichuan Academy of Medical Sciences and Sichuan Provincial People’s Hospital, School of Medicine, University of Electronic Science and Technology of China, Chengdu, China

**Keywords:** small-molecule inhibitors, epigenetic drugs, epigenetic reprogramming, cancer biomarker, histone modification, microRNA

## Abstract

Cancer treatment is a significant challenge for the global health system, although various pharmacological and therapeutic discoveries have been made. It has been widely established that cancer is associated with epigenetic modification, which is reversible and becomes an attractive target for drug development. Adding chemical groups to the DNA backbone and modifying histone proteins impart distinct characteristics on chromatin architecture. This process is mediated by various enzymes modifying chromatin structures to achieve the diversity of epigenetic space and the intricacy in gene expression files. After decades of effort, epigenetic modification has represented the hallmarks of different cancer types, and the enzymes involved in this process have provided novel targets for antitumor therapy development. Epigenetic drugs show significant effects on both preclinical and clinical studies in which the target development and research offer a promising direction for cancer therapy. Here, we summarize the different types of epigenetic enzymes which target corresponding protein domains, emphasize DNA methylation, histone modifications, and microRNA-mediated cooperation with epigenetic modification, and highlight recent achievements in developing targets for epigenetic inhibitor therapy. This article reviews current anticancer small-molecule inhibitors targeting epigenetic modified enzymes and displays their performances in different stages of clinical trials. Future studies are further needed to address their off-target effects and cytotoxicity to improve their clinical translation.

## Introduction

Epigenetics is rising to prominence in molecular cell biology as an evolutionary mechanism by which external factors have intermediate-term effects on gene expression without changing the underlying genetic sequence (Rodenhiser and Mann, 2006; Handel et al., 2010). The epigenetic modification includes, but does not limit to, DNA methylation of cytosine–guanine base (CpG) motif and a wide range of histone modifications, including methylation, acetylation, phosphorylation, sumoylation, and ubiquitination (Esteller, 2007; Herranz and Esteller, 2007). Epigenetics is a significant driver of biological complexity and has a role in developing many diseases (Feinberg and Irizarry, 2010; Flavahan et al., 2017; Feinberg, 2018). For example, silencing of tumor suppressor genes or activation of oncogenes by DNA methylation or histone modifications contributes to the onset of a diversity of cancers (Gronbaek et al., 2007). To date, the most well-established therapeutic field of epigenetics is cancer, in which DNA methylation, histone modification, and abnormal expression of microRNA have all been linked to tumor development (Shi et al., 2003; Yen et al., 2016). In this review, we summarize the basic principles manipulating the abovementioned epigenetic pathways and highlight the evidence of the promising clinical and preclinical results using small-molecule inhibitors against chromatin regulators for cancer treatment.

### Epigenetic Modifications and Human Diseases

Epigenetics is one of the fastest developing fields in biology (Nepali and Liou, 2021). Recent achievements highlight the accelerated development of epigenetics, such as the definition of a human DNA methylome at single-nucleotide resolution, the various discoveries of histone variants and modifications, the study of the CpG island in the genome, and the progress of genome-wide nucleosome positioning maps (Baldi, 2019). It is necessary for the same genotype to raise numerous different phenotypes so that epigenetic marks can persist during the development and can be passed on to the offspring. The potential location of epigenetic marks includes DNA methylation, histone modification, and nucleosome location. They are the key to regulating gene and noncoding RNA expression (Miranda Furtado et al., 2019). As a result, the research of these mutations in epigenetic markers and epigenetic mechanisms associated with diseases has been launched. A comprehensive understanding of the epigenetic mechanisms, their interactions, and changes in health and disease has become an important research topic. The importance of epigenetics in maintaining normal development is reflected in that many diseases occur when the wrong epigenetic markers are introduced or added at the wrong time or place (Ganesan et al., 2019). It is displayed by searching the keyword “epigenetics” on PubMed; it displays that there were around 200 articles published in 1999, but more than 54, 00 in 2021. Thus far, efforts in epigenetic research have mainly focused on cancer, but as the field has grown, it has provided new insights into other types of diseases (Angarica and Del Sol, 2017; Wu et al., 2019). Considering the global incidence of obesity, it cannot be explained only by genetic factors, environmental factors are more likely to be the driving factors. Epigenetics is one of the essential mechanisms which link environmental factors with gene expression changes. Since the year of 2008, research on the role of epigenetics in T2D has begun to develop (Ling and Rönn, 2019). In 2013, an epigenetic association study of obesity indicated that the DNA methylation difference of obese subjects was greater than that of lean subjects. Moreover, this study identified some CpG sites associated with obesity. Also, it showed that both differential methylation and differential variability could predict obesity and the reliability is about 70% (Xu et al., 2013). With the breakthrough in technology, it is possible to initiate epigenomic analysis on a large scale (Li et al., 2016; Azangou-Khyavy et al., 2020). Dayeh et al. found altered DNA methylation of 1,649 CpG sites annotated to 843 genes in islets from 15 T2D cases versus 34 controls. Out of these genes, other 102 exhibited differential gene expression in the islets from T2D donors (Dayeh et al., 2014). CDKN1A, PDE7B, and SEPT9 belong to the genes with decreased DNA methylation and increased gene expression in T2D islets (Dayeh et al., 2014). Therefore, the development of epigenetics would provide an open field for the discovery of targets for prediction and therapeutics in human diseases (Portela and Esteller, 2010).

### Epigenetics and Cancer

Epigenetics participates in all stages of cancer development (Bates, 2020). Achievements of the Human Genome Project (HGP) have provided thousands of new targets in cancer treatment (International Cancer Genome et al., 2010). However, the HGP did not explain the difference in gene expression during cancer development. The effect of epigenetics in cancer has raised the attention of scientists (Park and Han, 2019). Genetic and epigenetic mutations participate in tumorigenesis and metastasis by controlling the interaction between tumor suppressor genes with oncogenes. In contrast to genetic mutations, epigenetic mutations regulated gene expression without changing the genome sequence (Nebbioso et al., 2018). The development of epigenetic research provides insight for cancer diagnosis, treatment, and improvement of drug resistance (Verma, 2015; Ponnusamy et al., 2020)**.** For instance, the promoter containing CpG islands of breast cancer cells was selectively hypermethylated to inactivation of tumor suppressor gene expression, such as cell cycle regulator (*p16* ^*INK4a*^ and *p14* ^*ARF*^), apoptotic regulator *(APC*, *HIC1,* and *TWIST*), and DNA repair genes (GSTP1, BRCA1, and MGMT)*.* These well-known tumor suppressor genes promote the development of breast cancer by changing various physiological functions of the cell due to promoter hypermethylated (Shukla et al., 2019). The nature of epigenetic modification is dynamic and reversible, which ensures a new epigenetic program and reprograms cells according to different conditions and provides other targets for designing antitumor drugs. Current animal models can only reflect the advanced stage of tumor growth but cannot reflect the early events. The breakthrough of epigenetics indicates that the dynamic and reversible nature of epigenetic plays a vital role in the early development of cancer. Common epigenetic factors induce tumor cells to reprogram and, thus, have pluripotency. Elucidating the relationship between reprogramming-related transcription factors and tumor epigenomes may help understand the molecular basis of regulating the cancer phenotype (Kim, 2020). In addition to cancer therapy, epigenetics also can serve as biomarkers for cancer diagnosis and risk assessment due to epigenetic changes before histopathological changes (Verma, 2015). Pancreatic ductal adenocarcinoma is usually diagnosed in the advanced stage without a little effective treatment strategy. The development of epigenetic markers is helpful for the early diagnosis of this tumor. Various methylation markers have been reported in pancreatic ductal adenocarcinoma, such as *p16*, *hMLH1* and *hMLH2*, and *cyclin D2* (Matsubayashi et al., 2003; Kumari et al., 2009; Kisiel et al., 2012). In the following sections, the main events involved in epigenetic regulation in cancer are discussed.

### DNA Methylation

**FIGURE 1:**
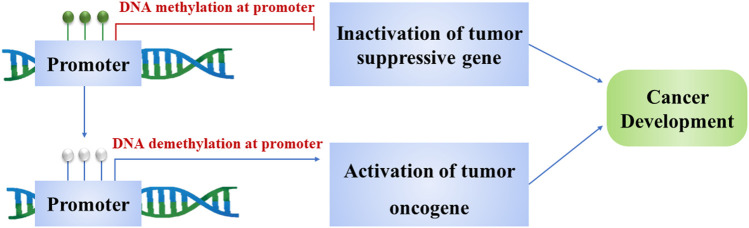
Methylation and demethylation of the gene promoter turn on tumorigenesis. Methylation of promoters inactivates tumor suppressor genes and induces cancer development. Demethylation of promoters activates oncogenes and results in the cancer cell proliferation.

DNA methylation is molecularly defined as a process that adds a methyl group to 5-carbon on cytosine residues (5mC) in CpG dinucleotides by DNA methyltransferase enzymes, which primarily exists in centromeres, telomeres, inactive X-chromosomes, and repeat sequences (Ning et al., 2016). The number of epigenetic modifications substantially outnumbers that of somatic mutations in human cancers. Also, individual tumor types can be stratified into subgroups based on different DNA methylation profiles (Pan et al., 2018). Consequently, DNA methylation has been regarded as a hallmark of cancer development and is characterized by global DNA hypomethylation of repetitive elements and CpG-poor regions concomitant with gene-specific DNA hypermethylation (Estecio and Issa, 2011; Liang and Weisenberger, 2017). Molecularly, DNA methylation alterations may lead to gene silencing due to DNA hypermethylation of CpG island promoter and gene activation owing to DNA hypomethylation of CpG-poor gene promoters, the process which is executed by DNA methyltransferases (DNMTs) (Figure 1) (Bestor and Verdine, 1994). DNMT1, DNMT3A, and DNMT3B are three well-established types of DNMTs responsible for maintaining chromosomal homeostasis (Zhang and Xu, 2017). Defective DNMTs may induce imbalance in DNA, which leads to the onset of chromatin remodeling, genomic instability, and gene inactivation (Nephew and Huang, 2003; Esteller, 2007). Gaudet et al. have established that either deletion or reduction of DNMT1 can result in substantial genome-wide hypomethylation and chromosomal instability (Gaudet et al., 2003); Qu et al. have reported that hypomethylated CpG islands (CGIs) of the HOXB cluster found in acute myelocytic leukemia are highly associated with DNMT3A mutations (Qu et al., 2014). These discoveries shed light on cancer diagnosis and treatment, realizing the enormous potential of genomic methylation abnormalities in tumorigenesis.

### Covalent Histone Modifications

**FIGURE 2:**
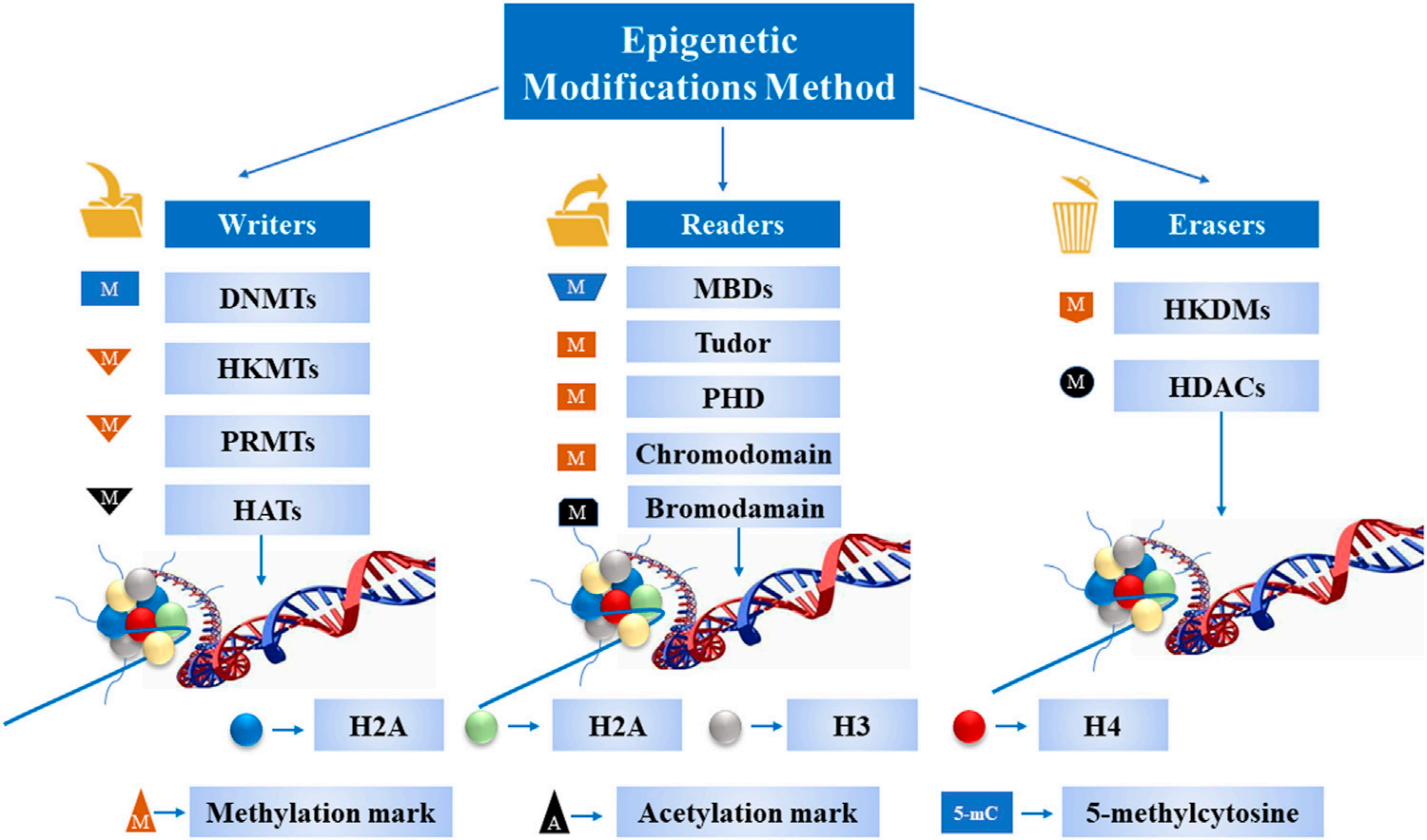
A schematic diagram of epigenetic tools. These enzymes and protein domains carry out most of the epigenetic modifications on DNA and histone tails.

Modulation of chromatin *via* covalent histone modification is one of the most fundamental ways to regulate DNA accessibility during physiological processes, including gene transcription, DNA replication, and DNA damage repair (Chi et al., 2010). To date, over ten different types of histone modifications have been identified to be involved in the process as mentioned above. The key modulators manipulating these modifications have been deciphered progressively with the better understanding of epigenetics. These modifications fall into three categories: *1*) writers: the enzymes are proficient in adding a nucleotide base and specific amino acid residues on histones; *2*) erasers: the enzymes are capable of removing a nucleotide base and specific amino acid residues; and *3*) readers: the proteins possess specialized domains that can recognize specific epigenetic marks in a locus. All these enzymes and protein domains are defined as epigenetic tools (Figure 2). The N-terminal tails of histones are usually the targets of covalent histone modification, which undergo a variety of posttranslational modifications, including methylation, acetylation, ubiquitylation, sumoylation, and phosphorylation on specific residues (Th'ng et al., 2005; Bhaumik et al., 2007). The establishment of an appropriate pattern of histone modifications is crucial for normal development and differentiation. On the contrary, the disorganized pattern of histone modification is associated with tumor initiation and development (Bannister and Kouzarides, 2011).

miRNAs are defined as small single-stranded noncoding RNA molecules (containing ∼22 nucleotides) found in mammals that function in gene silencing and posttranscriptional gene regulation (Hutvagner and Simard, 2008). Mechanically, miRNAs negatively regulate the gene expression of target mRNAs *via* the sequence-specific base pairing of miRNAs with 3′ untranslated regions of target messenger RNAs, followed by the cleavage of the mRNA strand (Hutvagner and Simard, 2008). Given the nature that miRNAs are expressed in a cell-specific manner and are involved in the safeguarding biological processes that include cell proliferation, differentiation, and apoptosis, aberrant miRNAs expression is involved in the cancers of different origins that include breast, colon, gastric, lung, prostate, and thyroid (Di Leva and Croce, 2013; Reddy, 2015). Unlike normal mRNAs regulated by epigenetic mechanisms, a tight connection occurs between miRNAs and epigenetic modification. On the one hand, epigenetic modification could result in the aberrancies of the miRNome (Valeri et al., 2009). The dysregulation of miRNome is defined as the hallmark of cancer initiation and metastasis. The majority of epigenetic regulation events are involved in the dysregulation of miRNome (Humphries et al., 2019). On the other hand, a specific group of miRNAs that is called epi-miRNAs can manipulate epigenetic regulatory mechanisms inside a cell by targeting enzymes that are responsible for DNA methylation (DNMT3A and DNMT3B) and histone modifications (EZH2) (Liu et al., 2013). MiRNA and epigenetics are feedback loops rather than liners (Yao et al., 2019). A primary theory has been established that miRNAs modulate epigenetics *via* regulating epigenetic modifier enzymes, which facilitate a trilateral regulatory “epi–miR–epi” feedback circuit in pathological and physiological processes. The result of this “epi–miR–epi” interaction has emerged as a new layer of complexity in gene regulation, whose comprehension sheds light on understanding human cancerogenesis.

#### Epigenetic Therapy of Cancer

The reversible nature of the profound epigenetic modification in cancer has raised the possibility of “epigenetic therapy” as a treatment option against refractory cancers. Several small-molecule inhibitors working as chromatin regulators have been at advanced stages of clinical trials, and the US Food and Drug Administration (FDA) has approved azanucleosides targeting DNMTs, vorinostat targeting HDACs, and fedratinib targeting JAK2 for clinical treatment (Dzobo, 2019). This success indicates that the rationale of developing small-molecule inhibitors targeting epigenetic pathways may represent a novel therapeutic approach in the clinical setting. A successful clinical introduction of epigenetic inhibitors such as DNA methyltransferase inhibitors (DNMTis) and histone deacetylase inhibitors (HDACis) has been well established in treating hematological malignancies. These discoveries have opened new unexplored areas to understand the pathogenesis of cancer development and provided new targets for antitumor therapy development (Prachayasittikul et al., 2017; Gambacorta et al., 2019).

#### DNA Methyltransferase Inhibitors

Tumor suppressor genes function mainly to either repress or inhibit the cell cycle or promote apoptosis (Joyce et al., 2021). The better-known tumor suppressor gene includes gene cyclin-dependent kinase inhibitor 2A (CDKN2A) (Zhao et al., 2016), breast cancer susceptibility gene breast cancer 1 (BRCA1) (Krais and Johnson, 2020), and adenomatous polyposis coli (APC) (Schrock et al., 2020). Global DNA hypomethylation and hypermethylation of the promoter regions of the tumor suppressor gene manipulated by DNMTs have been widely found in the malignant cells, which provide a promising target to develop drugs against DNMTs (Subramaniam et al., 2014). DNMTi includes two categories: nucleoside and nonnucleoside inhibitors (Singh et al., 2013). Among these small-molecule inhibitors, cytosine analogs azacytidine (5-azacytidine) and decitabine (5-aza-2′-deoxycytidine) are the two best known nucleosides DNMTis (Fahy et al., 2012). Molecularly, 5-azacytidine is an inducer of chromosome breakage and a mutagen by demonstrating its ability to incorporate itself into the human genome *via* various mechanisms (Imanishi et al., 2014). Its strategies include inhibition of tRNA methyltransferases, interference with tRNA methylation, and interruption of ribosomal RNA processing (Lu and Randerath, 1980; Grosso and Pitot, 1984; Mohana Kumar et al., 2006). In addition, 5-azacytidine can also interfere with de novo thymidylate synthesis, empowering its cytotoxicity effect (Osorio-Montalvo et al., 2018). Pharmacologically, 5-azacytidine and 5-aza-2′-deoxycytidine form an irreversible complex with the DNMTs, which results in the degradation of DNMTs (Valdez et al., 2010). To date, both drugs have been approved for the treatment of myelodysplastic syndrome (MDS) and AML in the clinical setting (Soriano et al., 2007). However, recognized by the pleiotropic effects of 5-azacytidine, 5-aza-2′-deoxycytidine, and their targets as mentioned above, researchers have confronted enormous challenges discovering novel inhibitors that are somewhat held back. To alleviate the toxic profiles of 5-azacytidine and 5-aza-2′-deoxycytidine, a less poisonous cytidine analog was developed, called zebularine. It exerts demethylation activity by stabilizing the binding of DNMTs to DNA, hindering the methylation and decreasing the dissociation, thereby trapping the enzyme and preventing turnover even at other sites (Sanaei and Kavoosi, 2020). It also enhances tumor cell chemo- and radiosensitivity and has antimitogenic and angiostatic activities (Balch et al., 2005; Hellebrekers et al., 2006; Greer et al., 2017). Zebularine inhibits DNA methylation and reactivates a gene previously silenced by methylation (Cheng et al., 2003). The mechanism of action of Zebularine is concentration dependent. High doses of Zebularine can induce cell cytotoxicity through double-strand breaks, cell cycle arrest, and causing DNA damage (Orta et al., 2017). Unfortunately, the high dose required for therapeutic value excluded Zebularine for its clinical application. Nowadays, there are three candidates from second-generation nucleoside DNMTi under clinical trials. SGI-110 is designed for the treatment of advanced hepatocellular carcinoma (NCT01752933), MDS and AML (NCT01261312), while 4′-thio-2′-deoxycytidine and RX-3117 are still under investigation against advanced solid tumors (NCT02423057) and metastatic pancreatic cancer (NCT03189914), respectively (Issa et al., 2015; Lu et al., 2020) (Campbell and Tummino, 2014).

Unlike the nucleoside analogs, nonnucleoside DNMTis directly bind to the catalytic region of DNMTs instead of incorporation into DNA. Consequently, the cytotoxicity of 5-azacytidine and 5-aza-2′-deoxycytidine is less than that of nucleoside DNMTis (Rondelet et al., 2017). 5-Azacytidine and 5-aza-2'-deoxycytidine are potent inhibitors of DNA methyltransferase. Its cytotoxicity has been attributed to several possible mechanisms, including reexpression of growth suppressor genes and formation of covalent adducts between DNA methyltransferase and 5-aza-2'-deoxycytidine–substituted DNA which may lead to steric inhibition of DNA function (Christman et al., 1985; Kiianitsa et al., 2020; Nunes et al., 2020). They include procainamide, procaine, epigallocatechin-3-gallate (EGCG), SGI-1027, nanaomycin A, flavonoid, and compound 5. Pharmacologically, procainamide and procaine can modify the CpG regions of DNA, resulting in blocking DNMTs activities (Li et al., 2018); Morris et al. reported that flavonoid and EGCG could inhibit DNMT1 enzyme activity from restoring RXRα expression in human colon cancer cells (Li et al., 2018). Datta et al. found that SGI-1027 (a quinoline derivative) could make the MLH1 and P16 promoter region in colon cancer cells reactive *via* inhibiting all three DNMTs (Datta et al., 2009). Similarly, it is documented that nanaomycin A can selectively target DNMT3a to induce the activation of tumor suppressor genes in cancer cell lines (Kuck et al., 2010). Compound 5 derived from a chemical modification of SGI-1027 is the first nonnucleoside DNMTi that has been investigated in cancer cell lines. It can display potent antiproliferative effects against histiocytic lymphoma, breast cancer, Burkitt’s lymphoma, and prostate cancer at micromolar doses (Zhou et al., 2018). However, few of these inhibitors have been used in the clinical setting owing to their dissatisfactory clinical safety and efficacy. Table 1 summarizes several drugs that are in the different stages of clinical trials.

**TABLE 1:** A list of DNA methyltransferase inhibitors under different phases of clinical trial and their indication.

DNA methyltransferase inhibitors				
Classification	Compounds	Structure	Clinical stage	References
Nucleoside analogue	Azacytidine	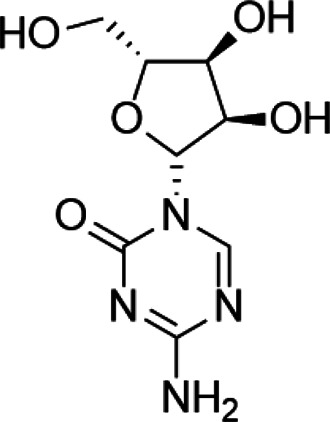	Phase III	Derissen et al. (2013)
	Decitabine	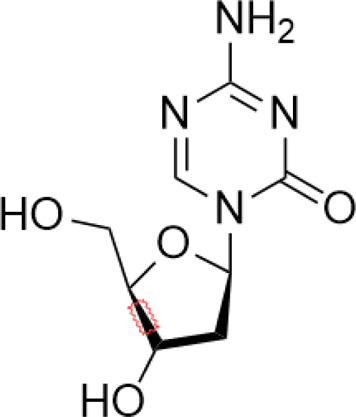	Phase II	Dhillon (2020)
		SGI-110	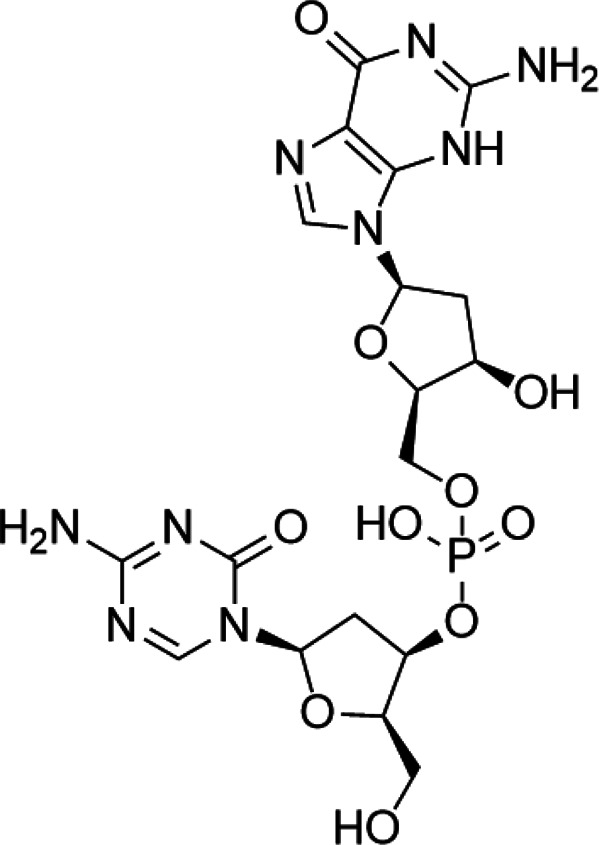	Phase II	(Daher-Reyes et al., 2019)
	Nonnucleoside analogue	Nanaomycin A	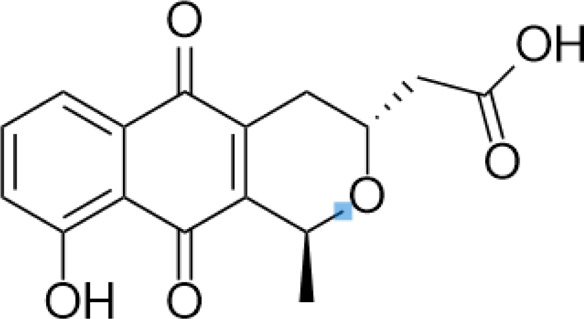	Preclinical	Nakamae et al. (2018)
SGI-1027		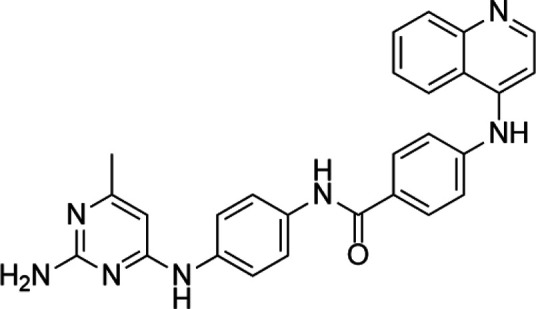	Preclinical	Sun et al. (2018)
		MG98	uncovered	Phase II	Linnekamp et al. (2017)

Lysine can be monomethylated, demethylated, or trimethylated by lysine methyltransferases (KMTs). Suv39h1 is the first histone KMT, and its main methylation site is H3K9. However, it was found that the site of H3K9 was almost no longer enzymatically active after the modification and its trimethylated peptide was no longer used as the substrate of methylase. In addition, the acetylation of H3K9 can inhibit the methylation of this site, and the dephosphorylation of H3S10 is the prerequisite for the methylation of H3K9. Thus, the phosphorylation of H3S10 can inhibit the methylation of adjacent site K9.

According to the types of amino acids at the modification sites, histone methylation can activate or inhibit gene transcription. For example, the methylation of histone H3K4, H3K36, and H3K79 sites can effectively activate the expression of corresponding genes, while the demethylation or trimethylation of H3K9, H3K27, and h4k20 is usually associated with gene silencing.

Histone lysine methylation plays an important role in the construction and maintenance of heterochromatin and euchromatin regions. In summary, lysine methylation regulates protein function mainly through two mechanisms: on the one hand, it can interact with other forms of PTMs; on the other hand, it can regulate protein function by influencing protein–protein interaction. Given that specific lysine residues on histone protein are prone to methylation, which subsequently leads to tumor development, researchers developed specific inhibitors to interfere with the catalytic activity of methyltransferases on histone protein methylation (Rea et al., 2000). These methyltransferases specific for H3K4 include SET1, MLL, and SMYD1&3 families of proteins (Biswas and Rao, 2018). Cao et al. reported that a small molecule (MM-401) could disrupt the methyltransferase activity of MLL1 (Cao et al., 2014). Methylation of H3K9 was executed by G9a, GLP, SETDB1/2, and SUV39H1/2 (Torrano et al., 2019). Chaetocin was initially designed as an HKMT inhibitor under this category (Sak et al., 2021). Since then, several modified inhibitors were developed. BIX-01294, the first selective inhibitor of G9a, and its advanced alternative UNC0638 are potential candidates as antitumor agents (Pirola et al., 2018). Pappano et al. reported that A-366, a peptide-competitive inhibitor of G9a and GLP, plays a key role in inhibiting leukemic cells (Pappano et al., 2015). Coincidentally, Yuan et al. also documented that BRD4770, an inhibitor of G9a, could induce pancreatic cancer cell death combined with gossypol (Yuan et al., 2013). As a well-established hallmark of cancer initiation, methylation of H3K27 is catalyzed by EZH1/2 (Dai et al., 2017). As a result, the inhibitors that target EZH1/2 have demonstrated promising effects on tumor shrinkage in the preclinical setting (Dai et al., 2017). It was reported that UNC 1999, a SAM-competitive dual inhibitor of EZH1/2, inhibited cell proliferation of MLL-rearranged acute leukemia (Yamagishi et al., 2019). Recently, constellation pharmaceuticals initiated a clinical trial for testing the safety and efficacy of CPI-1205, an EZH2 inhibitor against B-cell lymphoma (NCT02395601) (Gehling et al., 2015). Also, tazemetostat (an EZH2 inhibitor) is under clinical investigation (NCT03010982 and NCT03028103) (Kuntz et al., 2016). Methylation of H3K36 is another widely studied target for developing small-molecule inhibitors. Astra Zeneca identified AZ-505 as a specific inhibitor of methylation of H3K36 delayed cyst growth in a mouse model of polycystic kidney disease (Nguyen and Zhang, 2011). Nguyen et al. also reported that LLY-507, a selective inhibitor of SET and MYND domain-containing protein 2 (SMYD2) for methylation of H3K36, could abolish cell proliferation of several cancerous cell lines (Nguyen and Zhang, 2011). Besides, a disruptor of telomeric silencing 1-like (DOT1L), a histone H3K79 methyltransferase, has also been targeted to disrupt histone modification (Liu et al., 2014). Preclinical studies have demonstrated that DOT1L inhibitors that include EPZ004777, EPZ-5676, and SYC-522 can inhibit hematopoietic malignancies in different stages of clinical trials (Liu et al., 2014). Although most of the inhibitors mentioned above are still under clinical investigation, the recent accelerated approval of tazemetostat for metastatic or locally advanced epithelioid sarcoma sheds light on a promising direction towards further developing such compounds for cancer treatment. Table 2 lists several drugs that are currently in the preclinical and clinical trials.

**TABLE 2:** A list of histone lysine methyltransferase inhibitors under different phases of clinical trial and their indication.

Histone lysine methyltransferase inhibitors				
**Classification**	**Compounds**	**Structure**	**Clinical stage**	**References**
G9a (H3K9)	BIX-01294	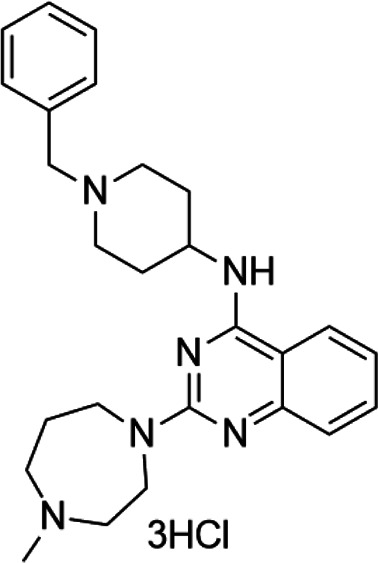	Preclinical	Deng et al. (2020a)
		UNC0638	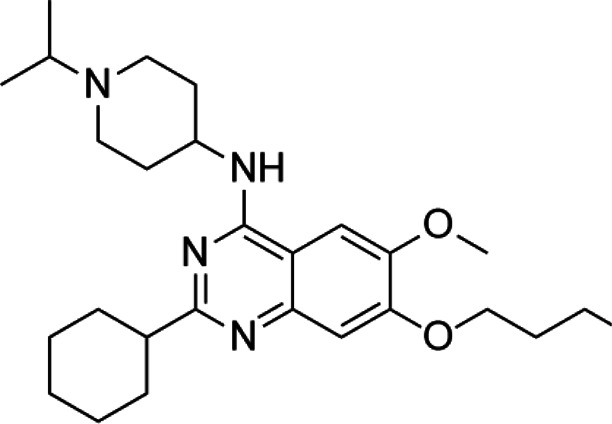	Preclinical	Nualkaew et al. (2020)
EZH2 (H3K27)	EI1	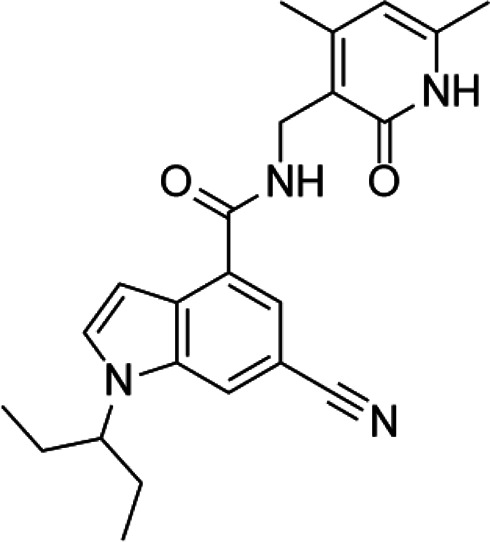	Preclinical	Fioravanti et al. (2018)
	CPI-1205	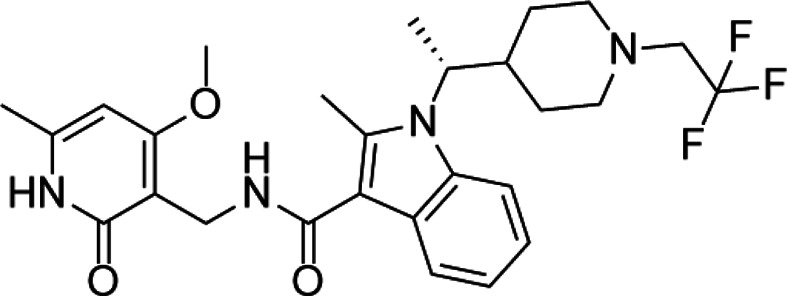	Clinical	Gulati et al. (2018)
		EPZ6438	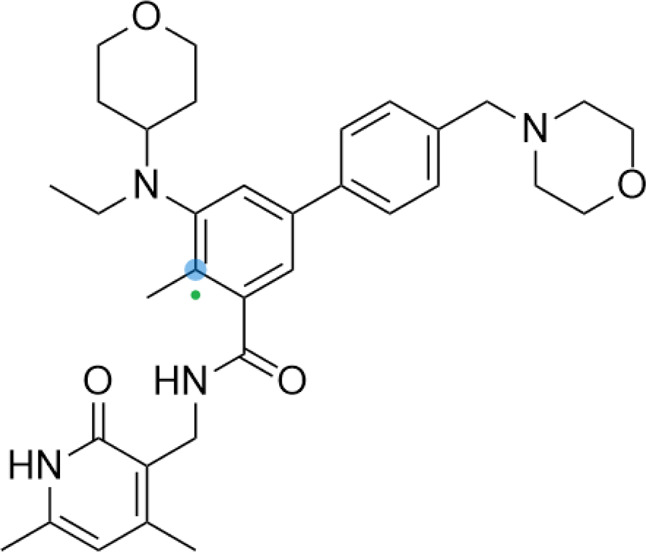	Clinical	Zhou et al. (2020)
	SMYD2 (H3K36)	AZ-505	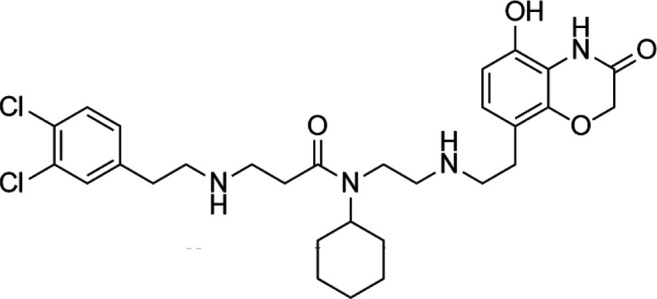	Preclinical	de Grass et al. (2014)
		LLY-507	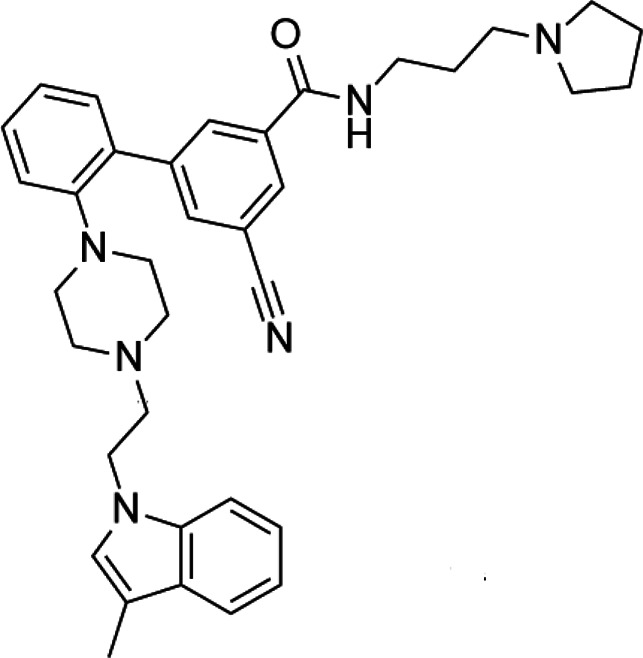	Preclinical	Kukita et al. (2019)
DOT1L (H3K79)	SYC-522	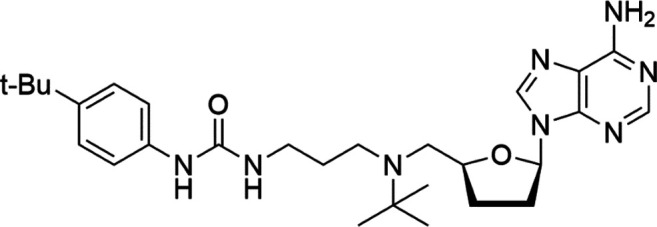	Preclinical	Liu et al. (2014)
		EPZ-5676	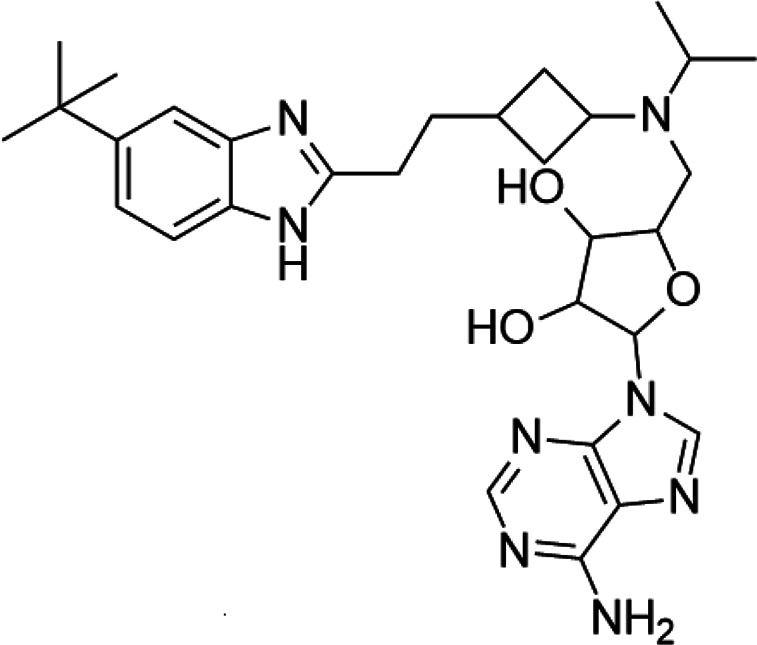	Clinical	

Methylation of histones can also occur in arginine residues, enabling the cell another layer of regulatory options (Cuthbert et al., 2004). Given the nature that arginine can be mono-, di-, or methylated modified, the modifications extend the complexity of gene regulation and are associated with transcriptional activation or suppression according to the location of the arginine residues (Wysocka et al., 2006; Jain and Clarke, 2019). Like the development of KDM inhibitors, the search for an arginine demethylase is also under active investigation. Physiologically, protein arginine methyltransferase (PRMT) can catalyze methylation of arginine residues on histones (Thompson and Fast, 2006; Jain and Clarke, 2019). PRMT family proteins and their arginine methylation are closely related to the occurrence and development of cancer. PRMT has nine members from PRMT1 to 9. Arginine methylation can be divided into monomethylation, symmetrical emethylation, and asymmetrical emethylation. According to arginine methylation, PRMT family members can be divided into three types: I, II, and III. Type I includes PRMT1, 2, 3, 4, 6, and 8, which can catalyze monomethylation and asymmetric dimethylation; type II includes Prmt5 and 9, which can catalyze monomethylation and symmetrical dimethylation; and type III includes prmt7 that can only catalyze monomethylation. Dysfunction of PRMT has been associated with different cancers, which leads to the efforts to developing specific inhibitors targeting this protein (Mohammad et al., 2019). Drew et al. reported that TP-064 and EZM2302, two inhibitors against PRMT4, inhibited the growth of multiple myeloma in the preclinical setting (Drew et al., 2017). EPZ015938, a selective inhibitor against PRMT5, is now under clinical investigation for patients with solid tumors and non-Hodgkin’s lymphoma (Siu et al., 2019). Bonday et al. also demonstrated that LLY-283, an inhibitor against PRMT5, can reduce tumor cell growth *in vitro* (Bonday et al., 2018). PRMT family members are often coexpressed and highly expressed in cancer, but its clinical significance is not clear. Liu Wen et al. confirmed that PRMT4, PRMT5, and PRMT7 were highly expressed in breast cancer, colorectal cancer, and prostate cancer, and the high expression of PRMTs was highly correlated with the enrichment of arginine methylation and abnormal alternative splicing of hnRNPA1. In breast cancer, colorectal cancer, and prostate cancer cells, PRMT4, PRMT5, and PRMT7 and their mediated hnRNPA1 methylation and splicing isomerism can effectively promote the growth of cancer cells. This provides a new direction and approach for cancer treatment. Table 3 highlights some PRMTis in the preclinical and clinical settings.

**TABLE 3:** A list of histone arginine methyltransferase inhibitors under different phases of clinical trial and their indication.

Histone arginine methyltransferase inhibitor				
**Classification**	**Compounds**	**Structure**	**Clinical stage**	**References**
PRMT1	DB75	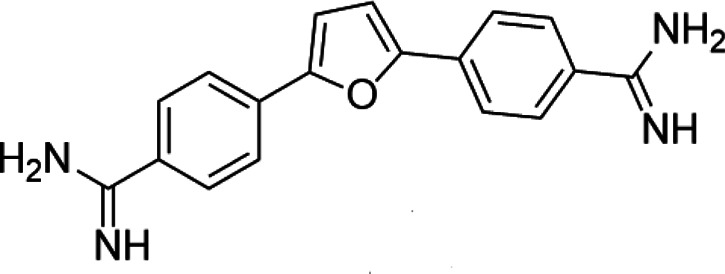	Preclinical	Oudard et al. (2017)
PRMT4	TP064	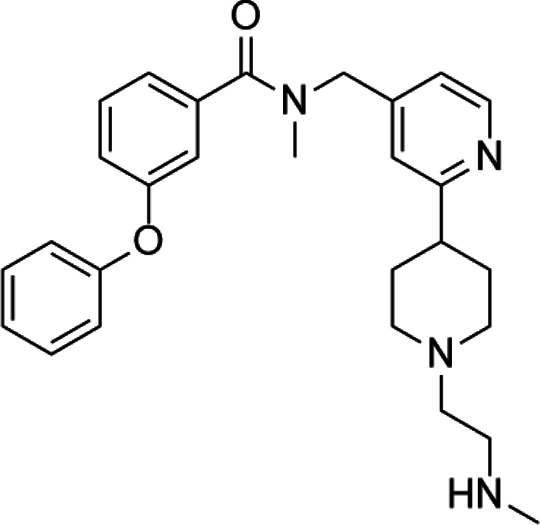	Preclinical	Loe et al. (2021)
PRMT5	EPZ015938 (GSK3326595)	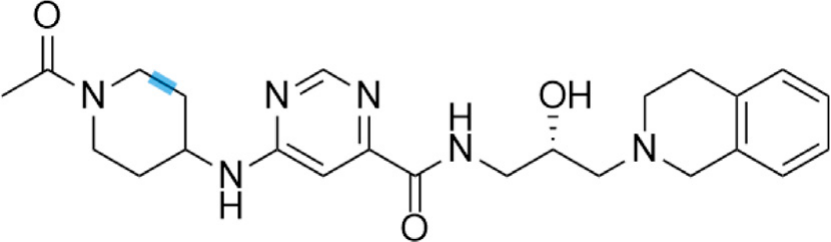	Clinical	Li et al. (2019)

Demethylation of lysine residues on histone proteins *via* targeting histone lysine demethylase KDM1 (LSD1/2) and KDM2-8 (JmjC domain proteins) represents another strategy of developing small-molecule inhibitors (Rotili and Mai, 2011; Hoffmann et al., 2012). Both families have been investigated for the development of inhibitors owing to their crucial role in tumorigenesis. Prusevich et al. found that bizine, the second generation of LSD1/2, significantly inhibited cancer cell proliferation *in vitro* (Prusevich et al., 2014). Zhu et al. reported that the inhibition of LSD1 reduced the growth of human breast cancer cell lines (Zhu et al., 2012). Willmann et al. demonstrated that LSD1 inhibition could also be used for androgen-dependent prostate cancer treatment (Willmann et al., 2012). Gupta recently identified that an irreversible LSD1 inhibitor, HCI-2509, was beneficial against MYCN-amplified neuroblastoma cells (Gupta et al., 2018). On the other hand, the development of specific inhibitors of JmjC-KDM also has come a long way. It was established that hydroxamic acid scaffold, hydroxyquinoline analogs, and cyclic peptides showed potential effectiveness as JmiC-KDM inhibitors (Rose et al., 2008). Though these inhibitors are still at the early stages of development, Hopkinson et al. and Thinnes et al. have initiated two preclinical studies to test the effectiveness of IOX1 and flavonoids against cancer cells (Hopkinson et al., 2013; Thinnes et al., 2014). Current drug development is summarized in Table 4.

**TABLE 4:** A list of histone demethylase inhibitors under different phases of clinical trial and their indication.

Histone demethylase inhibitor				
**Classification**	**Compounds**	**Structure**	**Clinical stage**	**References**
LSD1 inhibitors	Tranylcypromine analogue (GSK2879552)	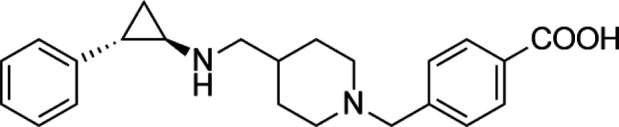	Clinical	Kalin et al. (2018)
	Bizine	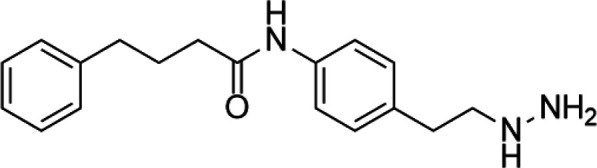	Preclinical	Kim et al. (2020)
	PG11144	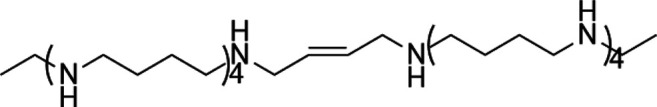	Preclinical	Zhu et al. (2012)
		Namoline	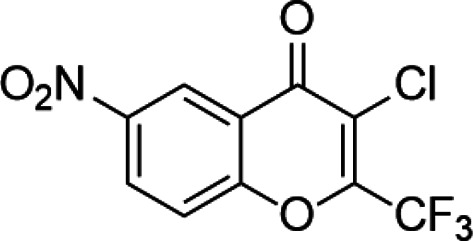	Preclinical	Sewry et al. (2019)
	JmjC domain inhibitors	IOX1	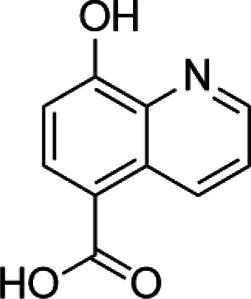	Preclinical	Hoyle et al. (2021)
			KDM6B	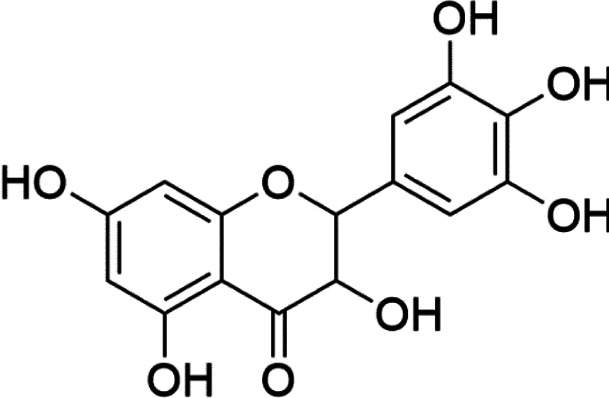	Preclinical	Basu Mallik et al. (2017)

Acetylation is a very common posttranslational modification (Drazic et al., 2016). In human cells, more than 1,750 proteins can be acetylated at lysine residues. Histone deacetylases (HDACs), as epigenetic modifiers, play an important role in gene transcription (Autin et al., 2019). Changes, mutations, and/or inappropriate recruitment of HDACs have been widely found, which are involved in tumorigenesis through a series of biological pathways (Hadley et al., 2019). Therefore, HDACs are considered a promising tumor therapeutic target, and their inhibitors are developing rapidly (Luparello et al., 2020). The application of HDAC inhibitors (HDACis) as anticancer drugs in cancer has been confirmed in cell lines and animal models (Falkenberg and Johnstone, 2014). The first generation of HDACi was developed based on screening by experience for some agents whose potential targets were HDACs. These agents originated from the tumor cell differentiation inducer, including butyrate, trichostatin A (TSA), and vorinostat (Leder and Leder, 1975; Riggs et al., 1977). Then, more HDACis were discovered from natural products, which have different properties and clinical settings (Johnstone, 2002; West and Johnstone, 2014). However, the traditional HDACi was targetting multiple HDACs, which led to the difficulty of verifying the biological consequences and toxicities from inhibition of a specific HDAC or/and combined effect of multiprotein HDAC complexes (Bantscheff et al., 2011). Therefore, researchers need to identify more molecules as a new HDACi generation with an improved activity and specificity. HDACi has four major classic structures, including hydroxamic acid derivatives, aminobenzamide, cyclic peptide, and short-chain fatty acids (Cappellacci et al., 2020). Then, we sum up the HDACi which has been approved by the FDA of USA in Table 5. More interestingly, advantages of multitargeting antitumor drugs have been presented due to the multifactorial nature of tumor etiology in this respect because histone deacetylase inhibitors play an important role in many anticancer activities and have become a privileged tool for the development of mixed drugs. EGFR/HER2/HDAC hybrid inhibitor CUDC-101 is the first success of multitargeting drugs, which is one of the HDAC/kinase dual-acting compounds family (Luan et al., 2019). The other excellent “hybrid drug” is PI3Ks/HDAC hybrid inhibitor CUDC-907, which entered a phase 2 clinical trial (Hesham et al., 2018). GUDC-101 and CUDC-907 exhibit improved synergistic effects than the single-targeted drugs and overcome resistance to receptor tyrosine kinase inhibitors *via* multiple signaling. HDAC/CDK-4/JAK1i and LSD1/HDACi are the novel multitargets to develop “hybrid drug.” Preclinical data of Roxyl-zhc-84 (HDAC/CDK-4/JAK1i) and corin (LSD1/HDACi) also show better therapeutic effect than single acting compounds alone or in combination (Huang et al., 2018; Kalin et al., 2018). The clinical and preclinical results of the abovementioned agents show that the development of high-efficiency multitarget hybrid drugs is worthy of further research. The design guideline of hybrid HDACi should keep the potency and drug similarity of single target compounds to their respective targets and have an acceptable ADMET spectrum, while avoiding the increased toxicity and targeting effect due to the decreased targeting selectivity.

**TABLE 5:** A list of histone deacetylase inhibitors under different phases of clinical trial and their indication.

Histone acetyltransferase inhibitors				
Classification	Compounds	Structure	Clinical stage	References
HDAC1/2i	MRLB-223	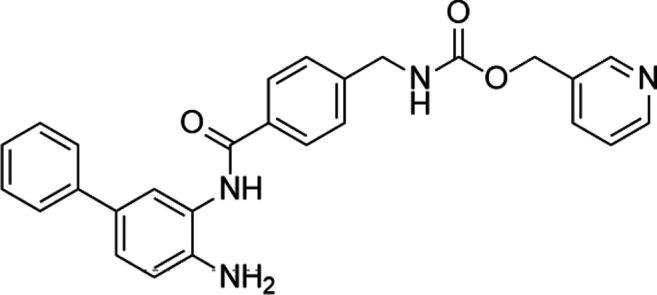	Preclinical	Newbold et al. (2013)
HDAC3i	BG45	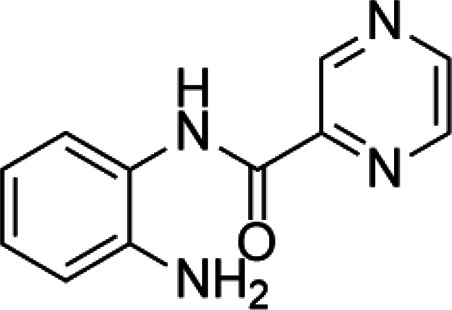	Preclinical	Tang et al. (2018)
HDAC6i	Rocilinostat (ACY-1215)	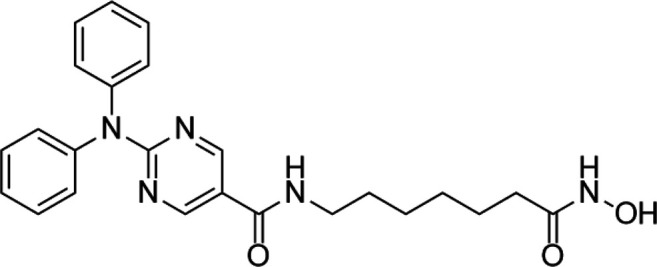	clinical	Yang et al. (2020)
	Tubacin	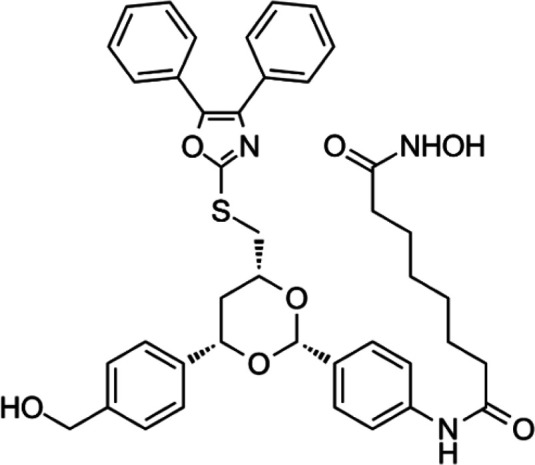	Preclinical	Liang et al. (2019)
HDAC8i	C1A	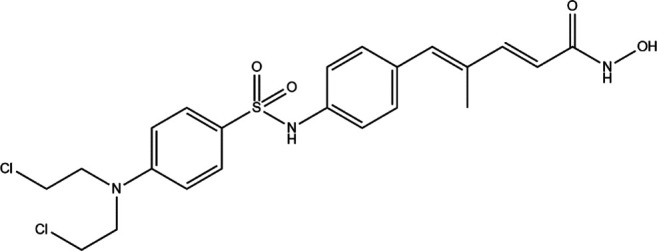	Preclinical	Kaliszczak et al. (2013)
	HPOB	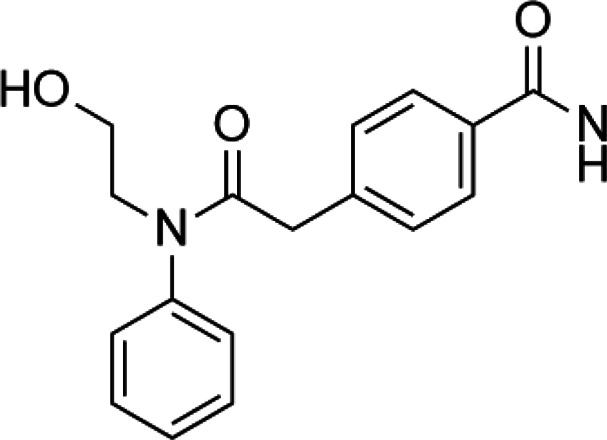	Preclinical	Liu et al. (2018)
	PCI-34051	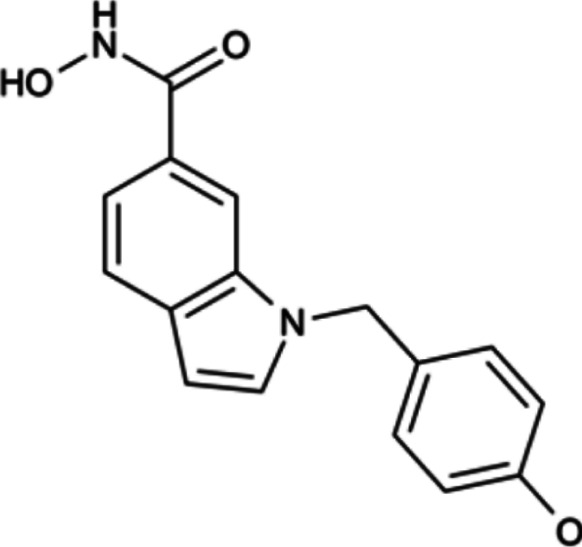	Preclinical	Morgen et al. (2020)
	C149	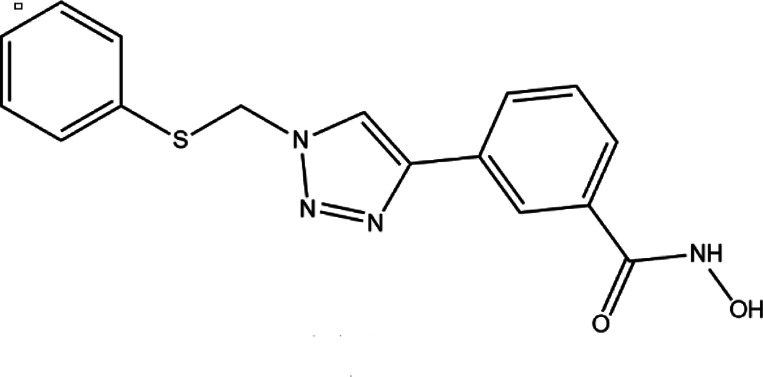	Preclinical	Suzuki et al. (2012)

Histone acetylation is considered as the best-studied histone modification, which occurs at the ɛ amino groups of evolutionarily conserved lysine residues on tail domains (Hake et al., 2004). From a functional perspective, histone acetylation is primarily associated with the activation of transcription. It mainly occurs at the regions of enhancers, promoters, and the gene body (Wang et al., 2009). Altered global levels of histone acetylation, such as acetylation of H4 at lysine (K), have been linked to tumor development in various cancers, which have also been found to be of potential prognostic value (Elsheikh et al., 2009). When hyperacetylation of proto-oncogenes occurs, the expression of the target genes will be activated. On the contrary, when hypoacetylation of tumor suppressors occurs, co-occurring with DNA methylation, the tumor suppressors will be inactivated. These two mechanisms collectively contribute to the onset of tumor initiation and development. The enzymes that catalyze the addition of acetyl groups to histone lysine residues are histone acetyltransferases (HATs) (Seto and Yoshida, 2014). Numerous chemical compounds have been tested for their potential as HAT inhibitors (HATis) (Carrozza et al., 2003; Eliseeva et al., 2007; Arif et al., 2010). Stimson et al. reported that PCAF and p300 inhibitors, two isothiazolinone-based compounds, could inhibit cell proliferation of colon cancer cells (Stimson et al., 2005). More recently, Modak et al. found that embelin, a natural compound of hydroxybenzoquinone class, could block the activity of PCAF (Modak et al., 2013). Sun et al. reported that HAT Tip60 could sensitize tumor cells against ionizing radiation (Sun et al., 2006). Gao et al. reported that TH 1834, a novel version of Tip60 inhibitor, can induce cancer cell apoptosis (Gao et al., 2014). However, the questions are that these chemical compounds are moderately toxic towards humans (Bruserud et al., 2007; Subramanian et al., 2010; Deng H. et al., 2020). As a result, researchers are still working on screening the better candidates with low toxicity but high effectiveness. Table 6 summarized two drugs that are in the preclinical stage.

**TABLE 6:** A list of histone acetyltransferase inhibitors under different phases of clinical trial and their indication.

Histone acetyltransferase inhibitors				
Classify	Compounds	Structure	Clinical stage	References
Tip60	TH1834	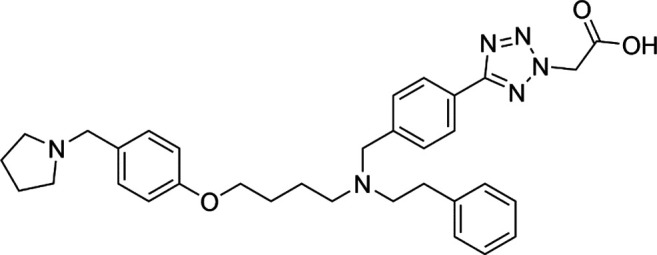	Preclinical	Idrissou et al. (2020)
p300	C646	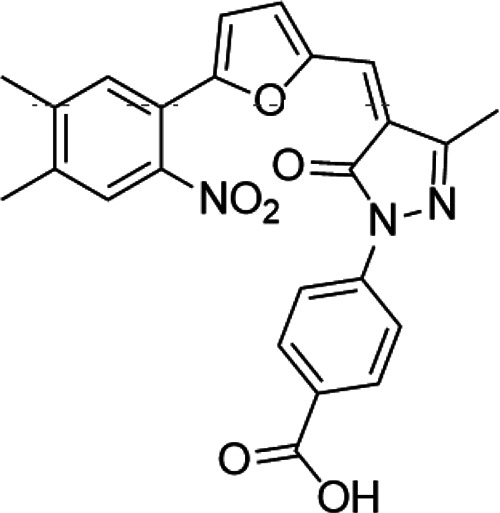	Preclinical	Ono et al. (2016)

### Small Molecules Targeting miRNAs

Given the well-documented nature that miRNAs play central roles in tumor development and because of the challenges of using nucleotide analogs for regulating miRNAs expression, it has been realized that the development of small-molecule drugs targeting specific miRNAs (SMIRs) would be a novel avenue for cancer treatment (Zhang et al., 2010). SMIRs are small synthetic organic molecules that can irreversibly bind to miRNAs. Mechanically, they bind to the grooves and pockets on the surface of miRNAs and interfere with the biological functions of targeted miRNAs (Mohammad et al., 2019). However, due to the structural flexibility and highly electronegative surfaces of SMIRs, RNA molecules have been excluded as drug target candidates for a long period. However, from the perspective of miRNA spatial structures, miRNAs appear to be “druggable” because the formation of stem loops in pre-miRNAs and the bulges in miRNAs can facilitate targeting by small molecules (Velagapudi et al., 2014). Of interest, Gumireddy et al. reported that diazobenzene and its derivatives could serve as specific inhibitors of pri-miR-21 formation (Gumireddy et al., 2008). Besides, it is documented that small-molecule enoxacin (Penetrex) can enhance small-interfering RNA-mediated mRNA silencing and facilitate the biogenesis of endogenous miRNAs (Shan et al., 2008). Though these mechanisms remain unclear, these findings undoubtedly provide proof of the modulation of miRNA activity by small-inhibitory molecules.

Recently, a novel strategy of developing a novel combined treatment therapy has been found. The rationale is rooted in the concept that many cancers share common gene or/and protein regulation pathways by chromatin regulators and miRNAs. For example, Swierczynski et al. comprised data from mirbase.org and DIANA-MICROT to find the overlap of HDAC-miRNA combinations. Then, they indicated HDACs and miRNAs shared some gene or/and protein regulation pathways (Swierczynski et al., 2015). Though detailed mechanisms remain to be elucidated, the complex linkage between miRNA and HDAC has emerged as a potential drug target, which might provide possible novel therapeutic approaches in the near future. It is reported that complete inhibition of HDAC2 can increase histone H4 pan-acetylation of the miR-183 promoter region and subsequently upregulate the transcriptional activity of miR-183, which leads to miR-183-mediated tumor suppression in neuroblastoma (Swierczynski et al., 2015; Zhu and Wang, 2021). Similarly, inhibition of HDAC3 with specific inhibitors can result in an increased hyperacetylation of the Dleu/miR-15a/16-1 promoter region. This upregulation increases the expression of miR-15a/16-1, which suppresses lung cancer cell growth. Besides, silencing of HDAC9 can stop sprouting *in vitro* and reduce vessel growth in a zebrafish model *in vivo via* the repression of the miR-17-92 cluster, indicating a possible common therapeutic target for cancer vasculogenesis (Hernandez-Romero et al., 2019). These could lead to personalized cancer therapies, which employ HDACs and simultaneously modify miRNAs. But, their mechanism of action remains to be addressed (Dawson and Kouzarides, 2012).

### Epigenetic Therapy (EpiDrugs) in Acquired Chemoresistance

Chemoresistance is a major obstacle to successful chemotherapy in clinic. Acquired drug resistance was controlled by multiple genetic and/or epigenetic ways (Ponnusamy et al., 2020). Unlike genetic mutations, epigenetic modulation in chemoresistance presents the characteristics of plasticity and reversibility, which puts a new insight into overcoming the acquired chemoresistance *via* epigenetic reprograming (Miranda Furtado et al., 2019). Recurrent tumors may still be sensitive to second-line chemotherapy because of the heterogeneity and poised epigenetics. However, during chemotherapy, the temporal epigenetic changes would induce acquired chemoresistance and lead to sensitive tumor no longer responding to second-line chemotherapy (Brown et al., 2014). The possible ways by which epigenetic dysregulation contribute to acquired chemoresistance are listed in detail as follows: *1*) Chemotherapy induces abnormalities in cell energy metabolism that regulate the generation/source of epigenetic factors and alter the cellular epigenetic spectrum, thereby promoting acquired chemoresistance (Wang et al., 2018); *2*) various efflux transporters, including p-glycoprotein, multidrug-resistant protein, and breast cancer resistance protein associated with acquired drug resistance showed epigenetic dysregulation during chemotherapy (Kim et al., 2014); *3*) epigenetic events such as DNA methylation and histone modification induce apoptotic tolerance and autophagy contributing to the development of acquired drug resistance (Hervouet et al., 2013; Sui et al., 2013); *4*) epigenetic dysregulation–mediated regulation of major tumor growth signaling and altered chemotherapeutic target expression may contribute to acquired chemoresistance; *5*) epigenetics also improves acquired chemoresistance by regulating genes involved in the formation of tumor microenvironments, such as tumor-associated fibroblasts and HIF-1α (Marks et al., 2016); *6*) cellular reprogramming regulated by epigenetic events was established and developed with acquired chemoresistance (Phi et al., 2018); *7*) epigenetic dysregulation and subsequent aberrant cellular energetics promote drug resistance by silencing genes involved in DNA repair or directly altering their structure (House et al., 2014). *8*) epigenetic dysregulation is a key pathway for ROS and its related oxidative stress to induce acquired chemoresistance (Shrishrimal et al., 2019). Therefore, it is not sufficient to target genetic abnormalities alone as a method to overcome acquired chemoresistance. Due to epigenetic heterogeneity in different patients and tumors, understanding the epigenetic dynamic landscape response to chemotherapy is necessary for EpiDrug discovery. Recently, clinical and preclinical studies have been conducted to evaluate the effect of EpiDrugs in overcoming drug resistance (Ediriweera et al., 2019). However, the results showed the double edges of EpiDrugs in chemoresistance. Due to the lack of specificity, despite EpiDrugs silencing tumor suppressors, they also hypomethylated microsatellite regions and activated oncogenes, promoting chemresistance (Ley et al., 2010). Taking together, developing EpiDrugs with a specific target and selectivity is critical and challenging. Meanwhile, dose adjustment and scheduling may be an important issue in EpiDrugs used to overcome chemoresistance (Ediriweera et al., 2019).

## Conclusion

Since the discovery of epigenetics by C. Waddington, tremendous development has been achieved in the field of epigenetics. Various enzymes and specialized proteins have been established for remodeling chromatin organization. Though cancer is a polygenic disease, studies have established a tight association of epigenetics with tumorigenesis. The profile of epigenetic alteration has provided novel targets for the development of antitumor agents as indicated by the US-FDA approval of HDAC inhibitors to treat a form of lymphoma (Giannini et al., 2012). However, enormous challenges remain to be overcome to accelerate the transition from bench to bedside. First of all, several substrates synergistically taking part in chromatin remodeling have been identified. In addition, most enzymes work as a part of a multiprotein complex, which increases the difficulty for active enzyme production and screening. These successful cases verify the hypothesis that it is possible to regulate the epigenetic process of treating diseases, and the therapeutic window of this new drug can be realized in the clinic. Although there are some ongoing clinical trials for a wide range of neoplastic and nonneoplastic diseases, the application of epigenetic drugs in clinical practice is mostly limited to hematological malignancies. The potential of epigenetic drugs is expanding to other diseases, from infectious diseases to brain diseases, cardiovascular diseases, and metabolic disorders. It seems promising, and more interesting results are expectant within a few years. However, the development of clinical trials needs to identify biomarkers that can predict drug response and avoid complications and unnecessary side effects in patients with nonsensitive tumors. Epigenetic mutations (hypermethylation of the tumor suppressor gene promoter) and epigenetic enzyme mutations (loss or gain of function) can be used as predictors of chemotherapy response in several cancers. For instance, epigenetic silencing of MGMT has been used as a biomarker to predict response to temozolomide in patients with glioblastoma. With the development of next-generation sequencing technology, it is possible to explore more unknown fields for the world. Therefore, further efforts will focus on increasing drug selectivity and expanding the spectrum towards solid tumors, since most of the clinically available epigenetic drugs are pan-HDAC inhibitors that are only effective against hematological malignancies. Appropriate patient selection and optimizing trial design and dosing schedules may also improve clinical efficacy.

## Abbreviations

AML, acute myeloid leukemia; CGIs, cytosine–guanine base islands; CpG, cytosine–guanine base; DNMTi, DNA methyl transferases inhibitors; DNMTs, DNA methyltransferases; DOT1L, disruptor of telomeric silencing 1-like; HATi, HAT inhibitors; HATs, histone acetyltransferases; 5mC, 5-carbon on cytosine residues; MDS, myelodysplastic syndrome; PRMT, protein arginine methyltransferase; and SMIRs, specific microRNAs.
